# Tertiary lymphoid structures: exploring opportunities to improve immunotherapy in ovarian cancer

**DOI:** 10.3389/fimmu.2025.1473969

**Published:** 2025-05-22

**Authors:** Aaron Varghese, Suzanne M. Hess, Shanmuga Chilakapati, Jose R. Conejo-Garcia, A.J. Robert McGray, Emese Zsiros

**Affiliations:** ^1^ Department of Gynecologic Oncology, Roswell Park Comprehensive Cancer Center, Buffalo, NY, United States; ^2^ Department of Obstetrics and Gynecology, University of Rochester Medical Center, Rochester, NY, United States; ^3^ Department of Pharmaceutical Sciences, Northeastern University, Boston, MA, United States; ^4^ Department of Integrative Immunobiology, Duke University School of Medicine, Durham, NC, United States; ^5^ Department of Immunology, Roswell Park Comprehensive Cancer Center, Buffalo, NY, United States

**Keywords:** tertiary lymphoid structures, ovarian cancer, immunotherapy, tumor microenvironment, gut microbiome, biomarkers

## Abstract

Tertiary lymphoid structures (TLS) are organized ectopic lymphoid clusters of immune cells that develop in non-lymphoid tissue to promote antigen presentation, drive cytotoxic immune responses, and enhance humoral immunity via B cell clonal expansion. Their presence within the tumor microenvironment (TME) correlates with increased patient survival and an improved response to immune checkpoint inhibitors (ICIs), positioning TLS as potential predictive and prognostic biomarkers. Despite the widespread use of ICIs across various cancers, their effectiveness remains limited in gynecological malignancies, including ovarian cancer (OC), a notably challenging disease characterized by poor responses to both single and combination ICI therapies. Interestingly, the infiltration of T cells into the OC TME is linked to enhanced progression-free survival (PFS) and overall survival (OS), yet an immunosuppressive TME frequently impedes therapeutic efficacy, suggesting cell activity within localized immune niches can impact antitumor immunity. This review explores the roles of TLS, their maturity, functionality, identification, and related gene signatures; specific immune cells and cytokines that play a role in TLS formation and antitumor response; and other modifiable elements, including gut microbiota, that may drive improving OC survival by leveraging a TLS-driven antitumor response to bolster immunotherapy outcomes.

## Introduction

Ovarian cancer (OC) represents the most lethal gynecologic malignancy in the United States, underscoring the need for innovative therapeutic strategies (1). OC is predominantly diagnosed at an advanced stage, where cytoreductive surgery and chemotherapy rarely produce curative benefit. The clinical trajectory for most patients is characterized by cycles of remission and relapse, with each remission period becoming progressively shorter until the disease develops resistance to chemotherapy or until significant toxicity arises (2). Given the limited clinical benefits of second line and subsequent therapies, a critical and ongoing need exists to develop novel therapeutic approaches.

Extensive evidence suggests OC is an immunogenic tumor that the host immune system can recognize. A higher infiltration of cytotoxic T cells within OC tumor islets is associated with significantly improved survival rates (3, 4). Tumor-specific T cell responses against multiple antigens overexpressed by OC, including folate receptor alpha (FRα), New York Esophageal Squamous Cell Carcinoma 1 (NY-ESO-1), p53, human epidermal growth factor receptor 2/neu (HER-2/neu), survivin, sperm surface protein 17 (Sp17), Wilms’ tumor 1 (WT1), transmembrane glycoprotein mucin 1 (MUC1), and melanoma-associated antigen-3 (MAGE-3), are quantifiable and highlight the potential for immunotherapy in treating OC (5–12). However, effective immunotherapy depends on the successful homing of functional tumor-specific cytotoxic T lymphocytes (CTLs) into tumors and chemotactic gradients within the tumor microenvironment (TME) (13–15) that support persistent immune cell infiltration or inflammatory function (8).

Even with the immunogenic nature of OC, immune checkpoint inhibitors (ICIs) show limited effectiveness in the relapsed/refractory setting, achieving response rates of only 8-15% (16–18). Currently, ICIs are approved solely for mismatch repair deficient (MMRd) OC or have high microsatellite instability (MSI) due to their inadequate effectiveness as single agents (19). While trials testing single agent antibodies targeting programmed cell death protein 1/program death-ligand 1(PD-1/PD-L1) or other ICIs in OC cancer patients have been disappointing (20), in a recent triple combination therapy clinical trial in advanced OC patients testing pembrolizumab, bevacizumab, and oral cyclophosphamide, one third of patients had a durable clinical benefit (DCB) (21–23). Comprehensive molecular, immunological, microbiome, and metabolic profiling analyses were performed on these patients’ biospecimens to assess response to this regimen. Increased T and B cell clusters and distinct microbial patterns with lipid and amino acid metabolism were linked to these patients with exceptional responses (23), compared to those with limited clinical benefit (LCB). Identifying reliable predictive biomarkers and who may benefit from immunotherapy would greatly contribute to patient selection for future immunotherapy clinical trials (21, 24–26).

### Immunosuppression in ovarian cancer

Despite successful CTL infiltration, tumor heterogeneity and an immunosuppressive microenvironment often undermine the antitumor immune response, with increased recruitment of immunosuppressive cells, including regulatory T cells (Tregs), tumor associated macrophages (TAMs) and myeloid derived stem cells (MDSCs), indicative of poor survival outcomes in OC (27–29). Low tumor mutational burden (TMB) and neoantigen (NA) load (30), downregulation of major histocompatibility complex (MHC)-1 in tumor cells (31), lysophosphatidic acid inhibition of type 1 interferon (32), an immunosuppressive environment in the ascites (33–36), immune evasion promoted by cancer driver mutations, including TP53 and phosphatase and tensin homolog deleted on chromosome ten (PTEN) (37), and aberrant oncogenic signaling pathways, all contribute to ICI resistance across various cancers (38). High metabolic demands of rapidly proliferating cancer cells also exhaust key nutrients needed for immune cell function. Elevated glycolysis rates in tumor cells lead to metabolic exhaustion in effector T cells, while lactate accumulation inhibits natural killer (NK) cell activation. Furthermore, increased expression of indoleamine-2,3-dioxygenase in tumor cells depletes tryptophan levels, impairing cytotoxic T cell proliferation, and inducing T cell exhaustion through kynurenine production (39–42).

Tumor-extrinsic factors also play a significant role in dampening antitumor immunity. Inadequate infiltration of lymphocytes into tumor islets is often due to abnormal vasculature and chemokine gradients (43), as well as compensatory mechanisms like upregulating inhibitory immune checkpoint receptor signaling, including cytotoxic T-lymphocyte associated protein 4 (CTLA-4) and lymphocyte activation gene 3 (LAG-3) (44, 45). Most recently, tertiary lymphoid structures (TLS) or organized ectopic lymphoid structures, that serve as hubs at sites of inflammation and support interactions between B and T lymphocytes and antigen-presenting cells (APCs) (46), has proved to be a major area of investigation (47). While the presence of TLS appears to be essential for coordinating robust antitumor responses, their absence impedes the integration of the humoral and cellular components of adaptive immunity, both of which have been deemed to be important for response to therapy. This limits immune activation and T-cell priming, thus underscoring the important role of TLS in effective tumor control. Given the critical role of TLS in enhancing antitumor immunity, this review will focus on presence and maturity of TLS, their significance on ICI efficacy and clinical trial outcomes, and the impact the gut microbiome and other factors have on antitumor immunity and TLS formation, especially in OC.

## Tertiary lymphoid structures

TLS support interactions where specialized formations of B cells, CD4^+^ and CD8^+^ T cells, and antigen presenting dendritic cells (DC) come together, along with high endothelial venule cells (HEV), in a coordinated manner, supported by a stromal infrastructure (48). TLS were initially described in autoimmune diseases, chronic infections, solid tumors, age-related diseases, and graft rejection (49–52), and have roles in local autoantibody formation, antibody-mediated immune responses in infected organs, and allograft rejection. TLS do not exist under physiological conditions which contrasts with primary (bone marrow and thymus) or secondary lymphoid organs (SLO). SLO are embryonic in nature and involve encapsulated lymph nodes (LN), the spleen, Peyer’s Patches, as well as tonsils, the human appendix, and mucosal-associated lymphoid tissues. SLOs are dependent on specialized Lymphoid Tissue inducer (LTi) cells that help to drive embryonic mesenchymal tissue organizing cells into follicular dendritic cells (FDC) and fibroblast reticular cells (FRC), driven by CXCL13 and CCL19/CCL21, respectively. TLS are generated after birth by specific cells involved in the immune response at sites of inflammation and are not associated with a specific organ. Both SLO and TLS are involved in generation of antigen-specific immune responses, antigen recognition, and activation of B and T cells (46), as well as involvement with HEV, specialized structures which help facilitate movement of lymphocytes from the blood into tissue. Due to TLS being sites or hubs of inflamed tissue without encapsulation, they are exposed to tumor antigens (TA), cytokines, and other inflammatory signals, which prompt a humoral and cellular immune response.

The presence of TLS correlate with favorable disease outcomes in a variety of cancers, while in autoimmune and chronic age-related disease, TLS are associated with worse, more severe outcomes (52). TLS formation has been observed in almost all organ specific human autoimmune diseases including Sjogren syndrome (SjS) (53), lupus nephritis (54), type I Diabetes (55), Crohn’s disease (56) and rheumatoid arthritis (57), however the prevalence of TLS is variable (52). Understanding the involvement of TLS in chronic diseases, including cancer, is therefore important regarding how they are formed and mature to determine potential strategies to aid in disease management.

The autoimmune SjS has been used as a model to derive a spatial and cellular map of key components involved in the formation and function of TLS. Single cell RNA, tissue transcriptomics, and spatial proteomics have been used on salivary glands from SjS patients (58). It has been shown that TLS formation and maturation including a Germinal Center (GC) correlates with autoimmunity, a pathological humoral response, B cell hyperactivity, and development of B cell lymphoma. Results from these studies suggest a complex cellular landscape of TLS, an immunomodulatory pericyte population in SjS, and significant fibroblast diversity, including presence of tissue-resident fibroblasts (immunofibroblasts) that have features like FRC in SLOs. There are different signals involved in the production of CCL21 and CCL19 cytokines in fibroblasts vs. pericytes, as well as distinct properties related to GC in SLO vs TLS. Transcriptomic and proteomic analysis between the SLO and TLS revealed differences in lymphoid structures and enrichment of certain cell types in mature TLS (mTLS), including genes involved in inflammatory cell recruitment, inflammatory pathways, and co-stimulation (58).

These specialized stroma derived fibroblasts have been determined to be crucial for the structure of TLS (59). Fibroblasts have been shown to exhibit plasticity and specialization under inflammatory conditions (48). While TLS are primarily composed of lymphocytes and DC, TLS are also supported by a complex network of additional stromal cells, including endothelial cells, lymphatic vessels, nerves, and immunofibroblasts. Immunofibroblast progenitors have been shown to be present at sites where TLS are established. A mouse model of TLS has shown that under chronic inflammatory conditions, tissue resident fibroblasts can acquire an immunofibroblast phenotype, including expression of lymphoid chemokines, adhesion molecules, and lymphocyte factors which sustain B and T cell survival in tissue. This process includes 1. priming by tumor necrosis factor (TNF) and interferon (INF) family members, interleukin (IL)-13, IL-1 family cytokines, IL-17, and IL-22, resulting in upregulation of intercellular adhesion molecule 1 (ICAM-1), vascular cell adhesion molecule (VCAM1), and podoplanin-posititve (PDPN); 2. expansion (fibroblast proliferation); and 3. maturation (stable expression of lymphoid cytokines CXCL13, CCL19, CCL21, and lymphocyte survival factors, IL-7 or B cell activating factor) of the immunofibroblast network (48). Upregulation of cell adhesion molecules-ICAM1 and VCAM1 also appear to facilitate network interactions.

**Figure 1:**
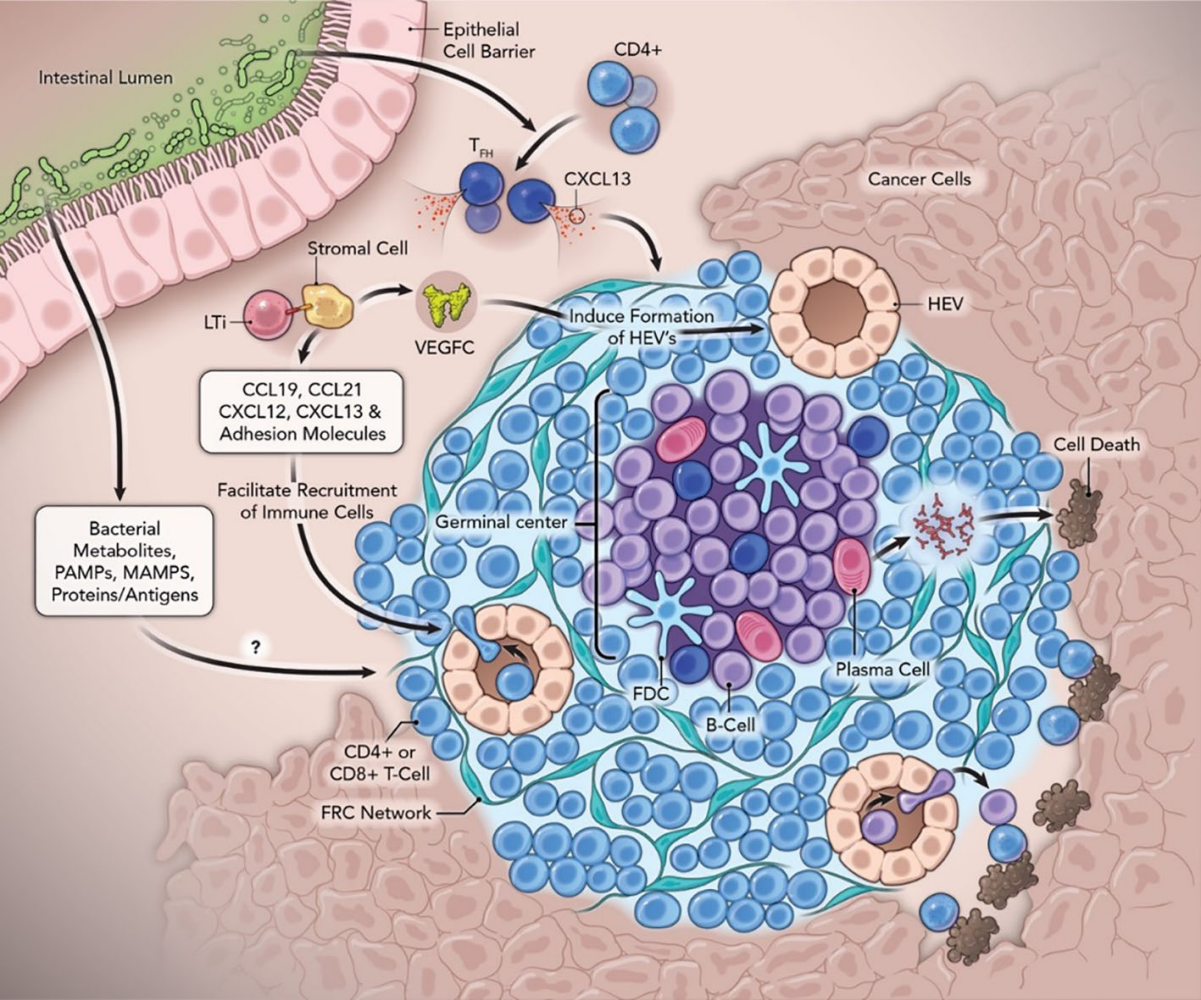
Neogenesis and function of tertiary lymphoid structures with the contribution of the gut microbiome in anti-cancer immunity. Abbreviations: FDC, follicular dendritic cells; Tfh, follicular helper T cells; FRC, fibroblastic reticular cells; GC, Germinal Centers; HEV, high endothelial venules; LTi, lymphoid tissue inducer cells; LTβR, lymphotoxin-β receptor; MAMPs, microbe-associated molecular patterns; PAMPs, pathogen-associated molecular patterns; PC, Plasma Cells; SHM, somatic hypermutation; TLS, tertiary lymphoid structure; VEGFC, vascular endothelial growth factor C.

A variety of cell types are associated with TLS (**Figure 1**). These organized structures are composed of B cell-containing GCs and DC-lysosome-associated membrane glycoprotein-positive dendritic cells (DC-LAMP)^+^ for antigen presentation (60). TLS are also marked by peripheral T cell zones with CD4^+^/CD8^+^/T^fc^ cells and peripheral node addressin (PNAd^+^) HEVs (60–62). B lymphocytes which play an active role, express activation-induced deaminase, an enzyme vital to class switch recombination and somatic hypermutation, which leads to the production of antigen-specific antibodies (63). Inflammatory factors are directly implicated in TLS formation and released from activated T cells, macrophages (MP), and DCs in TLS (64, 65).

### TLS maturity

TLS maturation stage is a key determinant of TLS function and mTLS have been linked to improved survival and sensitivity to ICI in several cancers. Discussions are ongoing regarding how TLS are defined, their maturity, their increased levels of organization and complexity, and the involvement of immune cells to drive antitumor immunity more effectively, compared to loose immune aggregates of T and B cells observed in immature or “early” TLS. (66–68). As such, the careful classification of TLS and how their presence/location can impact patient outcome or treatment response represents active areas of ongoing investigation. For example, it has been proposed that B cells that accumulate in immature TLS can develop into regulatory B cells with immunosuppressive features that support tumor progression. In contrast, B cells found in mTLS associated with GC are activated and proliferate, undergo affinity maturation, as well as isotype switching, resulting in plasma cells (PC), which produce tumor-specific antibodies, resulting in improved survival and immunotherapy response (66).

At least three levels of organization for TLS have been described: Lymphoid aggregates with minimal organization and occasional DCs; Immature DCs with an organized T and B cell presence, a network of FDCs, no GC, and possibly HEVs; and mTLS with active GC and HEV, and full B and T cell zones for activation of cell proliferation and recruitment of immune cells (69). Vanhersecke has proposed a standardized method to screen mTLS in cancer samples using hematoxylin-eosin-saffron (HES) staining and immunohistochemistry (IHC) that can be applied to all specimens (70). Vanhersecke has previously defined only two TLS categories, including mTLS (secondary follicle-like TLS) with TLSs containing either a visible GC on HES staining or CD23+ FDC or immature TLS (including early aggregation and primary follicle-like TLS) (68, 70). Of note, the clinical specimen used for TLS detection (surgical vs. biopsy vs. metastatic) had an impact, with TLS identification more prevalent in larger tissue samples.

In gynecological cancers, including OC, Zhang et al. (71) classified these heterogenous TLS into 3 different categories: a very diffuse group of lymphoid aggregate cells containing stomal, dendritic, memory B, follicular B and T cells, and CD4+ and CD8+ T cells; an immature TLS, with more organization including follicular B cells and a CD4+ and CD8+ T cell zone surrounding them with HEV at the periphery, and finally, mTLS consisting of mainly B cell follicles with GC in a B cell zone with networks of CD21+FDCs near an adjacent T cell zone of CD4+ and CD8+ cells, surrounded by stromal and fibroblast cells, PC, and HEV (71).

The organization and maturity of TLS also appears to play a significant role in response to ICI in OC. High-grade serous ovarian cancer (HGSOC) have a low density of follicular helper T (Tfh) cells resulting in a limited number of mTLS, with accumulation of TIM3+PD-1+, rather than TCF1+PD1+ CD8+ T cells, which may at least in part promote ICI resistance in HGSOC patients (72). The quality of TLS, i.e., how well they are formed, have also recently been implicated in OC relapse (73). In addition to changes in T cell localization and increased glycoprotein PDPN+ cancer associated fibroblasts (CAF), which help regulate tumor development and activity of immune cells, malformed TLS-like aggregates and some lymphoid aggregates were associated with spatial patterns of early OC relapse, with PC allocated into compartments associated with TLS-like aggregates and CAFs, potentially accounting for context-dependent roles for PC in HGSOC. In addition, PDPN+ CAFs were frequently associated with partially organized immune cells.

### TLS formation

It was thought TLS formation was similar to SLO induction involving LTi cells, hematopoietic cells with a critical immune function during embryonic development (74), which express lymphotoxin-α_1_β_2_ ligand and interact with the lymphotoxin-β receptor (LTβR) on lymphoid tissue organizer (LTo) cells to form aggregates. LTi cells interact with LTβR-expressing stromal cells, producing homeostatic chemokines, which are essential for an organization phase. TLS-associated cells are regulated during each phase of TLS formation by several categories of cytokines/chemokines that participate in stromal cell activation, LiTi cell aggregation, the loop between LTi and LTo, expansion of HEV and recruitment of lymphocytes, formation of T and B cell compartments, and GC formation with differentiation of B cells (63). These chemokines recruit B cells and T cells (via CXCL13, CCL19, CCL21) to form distinct immunological zones (75, 76). LTβR signaling also induces the production of VCAM-1, mucosal vascular addressin cell adhesion molecule-1, and ICAM-1, which further promote lymphangiogenesis (77).

It is now proposed T and B cells are surrogate LTi cells in TLS attracted to the inflammatory site by CXCL13 and IL-7 which activate LTo cells including stromal or immune cells via the TNF family receptor (78). LTo produce chemokines (CCL19 and CCL21 and CXCL10 and CXCL13) to attract immune cells near the site of immune activation and vascularization by establishing gradients which help guide the cells to the lymphoid structures. Adhesion molecules VCAM-1, ICAM-1, MAdCAM-1, and PNAd help circulating immune cells get from HEV to tissue and survival factors BAFF and IL-7 aid in B and T cell maturation and survival. It is thought that in OC, CD4+ T cells and DCs secrete cytokines as potential LTo cells (79).

In contrast to the pathway mentioned above, an alternative mechanistic pathway of lymphangiogenesis that generates TLS involves TNF superfamily member 14 (TNFSF14)/LIGHT, a cytokine produced by activated T cells (80, 81). LIGHT fused to a vascular targeting peptide has been found to normalize tumor blood vessels, activate CD4^+^/CD8^+^ T cells, and triggers TLS formation in immunotherapy-resistant pancreatic adenocarcinoma (64). Here, TLS were also found intratumorally instead of at the conventional tumor periphery, which could be due to vessel stabilization in deep tumor parenchyma and relocation of macrophages and effector T cells into the TME (64). In contrast, Tfh tumor-infiltrating lymphocytes (TILs), which secrete LIGHT, IL-21, and CXCL13, are sufficient to initiate TLS assembly, but at peritoneal tumor beds in OC murine models (82, 83)

Analysis of chemokine profiles in HGSOC supports the role of B cells in recruitment of DC-LAMP^+^, the latter which function in TA uptake and presentation for effective T cell priming (84). B cell clonal expansion correlates with an immunologic response in OC where CD20^+^ B cells that infiltrate tumors possess qualities of APCs, including surface expression of MHC class I/II, CD80, and CD86 (85, 86). Nielsen et al. suggested CD20^+^ TILs could serve as APCs to cytolytic CD8^+^ TILs, and the simultaneous presence of both B- and T-TIL subsets contribute to improved survival in OC patients (86). Montfort and colleagues demonstrated TLS present in OC omental specimens had an upregulated class-switched, memory B cell phenotype after neoadjuvant chemotherapy, suggesting that chemotherapy can regulate B cell functional status within TLS (84).

## Detection of TLS

Several methods, including pathological diagnosis, to detect, characterize, and quantify TLS as a predictive and prognostic biomarker have been used including H&E, multiplex IHC and immunofluorescence, and transcriptomic means, however, a consensus method has not yet been determined. While H&E staining is easy, affordable, and widely accepted, there are some concerns including potential for bias, reproducibility among pathologists, and underrepresentation of TLS structures (71). Several markers have been used to identify and quantify key immunological players in TLS through IHC with mTLS identified by markers of T cells (CD3, CD8), B cells (CD20), CD21(B cell), PNAd (HEV), CD208 (DC), CD79A (B cells), PC (CD138), proliferation (Ki67), and cytokines (CXCL13/BCL6). Early and immature phenotypes include CD20, CD3, CD79A, CD8, and PNAd markers, while aggregates include CD20 and CD3 and CD68 MP (87). Additional methods, including IHC and immunofluorescence, are used to identify TLS through staining methods and spatial detection and visualization, respectively, of target proteins in the TLS.

Newer approaches have been used to automate and detect TLS. Multi-resolution deep learning based on HookNet-TLS, has been shown to automate TLS quantification, and identify GC in H&E- stained digital pathology slides. This approach was recently shown to characterize TLS and their prognostic relevance in lung squamous cell carcinoma, muscle invasive bladder cancer, and clear cell renal cell carcinoma (ccRCC). While TCGA slides for OC were not initially used to develop this tool, its availability may provide opportunities to validate it in the OC patient population (88). Multi-plexed whole specimen tissue imaging, 3D reconstruction, spatial statistics, and machine learning was used in a colorectal cancer (CRC) model to determine relevant morphological features associated with diagnostic and prognostic significance, including TLS (89). TLS were commonly interconnected, formed larger 3D structures or TLS networks, had graded molecular properties, and were found in patients’ samples at various locations.

Identifying new ways to effectively image TLS in the TME will be invaluable in advancing this field of study and may involve the use of multiple spatial -omic technologies including spatial transcriptomics, proteomics, and metabolomics (90) to identify TLS. Imaging-based approaches will also be important for identifying spatial patterns of TLS that may be associated with early relapse, presence of CAFs (73), risk stratification (91) or assessing recurrence of cancers through flow cytometry and co-detection by indexing (CODEX) (92). Li et al. have suggested several biomaterials that may be suitable for monitoring TLS (93), while three-dimensional imaging may also be an option to characterize TLS (94). Positron emission tomography (PET) using18-F-fluoro-2-deoxy-D-glucose (18F-FDG)(PET/FDG) tail vein injections, combined with computed tomography (CT) for anatomical localization and single photon emission computed tomography (SPECT) by intraperitoneal injection of 99mTC labeled Albumin Nanocoll (99mTC-Nanocoll), has been explored in murine models of systemic lupus erythematosus (SLE) to detect early kidney changes and TLS presence. While TLS were detected in pancreas and not kidney, future studies using new PET/SPECT tracer administration sites, together with more specific tracers in combination with magnetic resonance imaging (MRI), may make it possible to detect formation of TLS and LN for pre-clinical studies. This may be relevant in OC due to different sites of origin of OC. Additional opportunities may include CT-based radiomic models to non-invasively predict intratumoral TLS (iTLS) as demonstrated for hepatocellular carcinoma (HCC) (95) and invasive pulmonary adenocarcinoma (96), as well as the transfer learning radiomic model, an MRI-based model to detect iTLS in HCC which was found to correlate with favorable prognosis and responsiveness to combination therapy in the context of higher model scores (97).

### Genomic signatures of TLS

Gene expression signatures have been used to identify TLS in a variety of cancers and determine their association to survival. A three-gene expression signature including IL-7, LTB, and CXCL13 was associated with LN neogenesis in human oral cancer, where higher grades of TLS were associated with improved disease-free survival (DFS) and overall survival (OS) (98). A unique 12-chemokine (CK) gene expression signature enriched for immune- and inflammation-related genes in primary CRC (99), identified TLS, and found an association with better patient survival independent of tumor staging. This gene signature has been used to confirm the presence of TLS in a variety of cancers including melanoma (100), breast (101), and bladder (102). A 12-gene signature associated with TLS derived from melanoma patient samples treated with ICI predicted clinical outcomes associated with B cells, immune cells, and CD8^+^ T cell-specific genes, including CXCL13 (103).

Recent OC studies have determined more involvement of TLS in immunotherapy responses. The presence of TLSs in OC and their potential clinical significance was also determined using the 12-CK signature (CCL2, CCL3, CCL4, CCL5, CCL8, CCL18, CCL19, CCL21, CXCL9, CXCL10, CXCL11, and CXCL13) previously identified (91). A 9-gene signature for TLS using a TCGA dataset was constructed and validated in the GSE140082 dataset (104). High expression of this gene signature (CETP, CCR7, SELL, LAMP3, CCL19, CXCL9, CXCL10, CXCL11, CXCL13) positively correlated with developed immune infiltration, and reduced immune escape with TLS associated with favorable responses to ICI in HGSOC patients. High-TLS clusters were characterized by better clinical prognosis, higher immune infiltration, more biological pathways, a higher TMB score, and higher expression of immune checkpoint (71). TLS strongly correlated with the immune-responsive microenvironment and were a favorable prognostic factor independent of other clinical characteristics. While the 12-gene CK signature associated with OC compares to TLS gene signatures from other cancers, additional genes appeared to be unique in the 9-gene signature, including CETP, CCR7, SELL, and LAMP 3. Using this signature, Lu et al. (105), showed TLS-high HGSOC tumors associated with better PFS, B cell maturation, and cytotoxic tumor-specific T cell activation and proliferation.

Recently, Chen et al. explored relevance of the 12-CK gene signature in gynecologic cancers and found it resonated most with cervical cancer, then endometrial cancer (EC), and finally OC (67). In addressing TLS in EC in biobanked samples from the PORTEC study, TLS associated with prognosis in only 2 of 4 EC subtypes: an *excellent* prognosis was associated with the ultra-mutated EC with DNA-polymerase epsilon exonuclease domain mutations (*POLE* mut), and an *intermediate* prognosis with hypermutated EC with MMRd (103, 106). TLSs were also found to assess recurrence with lower 5-year recurrence risk by 4-fold in EC patients (32.6% without TLS vs. 7.2% with TLS) (106). Mature TLS, presence of naïve-B, cycling/GC B cells, and antibody-secreting cells were also associated with L1CAM expression, a cell adhesion molecule independent of tumor expression, and what appears to be a specific biomarker for GC cells in mTLS. This might be a potential biomarker in OC, as well as EC. Note, in OC, TLS loss/dysfunction was linked to chromosome 4q deletion/DCAF15 amplification, whose copy number loss of IL15 and CXCL10 may limit TLS formation (105).

Overall, TLS related gene signatures may be relevant for OC patient stratification to different immunotherapies or responses. Specific gene signatures may not only predict the presence of TLS, involvement of immune markers, and clinical outcomes in different cancers, but may also select patients for specific immunotherapies based on molecular profiling of intratumoral lymphoid aggregates (65). Additional TLS-cancer-specific gene signatures may be forthcoming with artificial intelligence (AI)-driven assessment of large cancer-based datasets and further validation.

## Clinical value of TLS

TLS have been identified in a variety of tumor models with characterizations of pro- and anti-tumorigenicity (46), however, the potential for TLS to serve as predictive ICI-based biomarkers is strengthened through clinical trials of solid tumors assessing TLS in immunological, pathological, and clinically relevant outcomes (**Table 1**), and serve as a potential model for OC. While there is a limited amount of OC clinical trials performed where TLS has been accessed as a biomarker, several studies have shown promising results (**Table 2**), including those that also indicate the importance B cells in survival and the anti-tumor response (61, 63, 107, 108). Immune and stromal transcriptomic profiles for melanoma patients who respond to neoadjuvant ICI therapy reveal TLS clusters with the highest B cell signature have significantly longer survival (109, 110). Additionally, co-occurrence of tumor-associated CD8^+^ T cells and CD20^+^ B cells improves survival in metastatic melanoma patient samples, where TLS in CD8^+^CD20^+^ tumors stained for CXCL13, CXCR5, and CD20, and B cell-enriched tumors had increased TCF-7 naive and/or memory T cell levels (111).

**Table 1:** Relevant cancer clinical trials with tertiary lymphoid structure involvement in outcomes.

Author, Year, [Ref]	Cancer Type	Clinical Trial NCT Number Phase	Patients	Therapy	Endpoint	Outcomes and TLS Involvement
Garaud et al., 2018 (169)	Breast	N/A	66 primary BC patients.	91-Ag microarray, IgG & IgA.	Analysis of autoantibodies & tumor features.	ex vivo IgA autoantibodies only reactive to BC-associated Ag. Linked with GC and early memory B cell maturation & TLS, suggesting TIL-B activated in the TME.
Maldonado et al., 2014 (142)	Cervical	NCT00788164 Phase 1	12 patients with HPV 16+ CIN.	IM vaccine targeting HPV16 E6/E7.	Safety, tolerability, feasibility, immuno-genicity. Dose and side effect efficacy +/- imiquimod.	Post-vac cervical tissue immune infiltrates included organized stromal TLLS. Increased expression of genes associated with immune activation (CXCR3), effector function (Tbet & IFNβ) & immunologic signature in the overlying dysplastic epithelium. TCR-seq of unmanipulated specimens identified clonal expansions in tissue not detectable in peripheral blood. Vaccination to HPV antigens can induce a robust tissue-localized effector immune response.
Horeweg et al., 2022 (106)	Endo-metrioid	NCT00411138 PORTEC 3 Phase 3	Stage IA G3/II-III endometrioid or stage I-III serous CC endometrial AC.	Chemo XRT + 4 C chemo vs XRT.	Primary outcome, OS, failure free survival. 411 EC slides analyzed.	Chemo XRT + Chemo no improved OS. TLS associated in POLE hypermutated and MMRd & is a prognostic factor. L1CAM identified as a potential TLS biomarker.
Ho et al., 2021 (120)	Hepato-cellular carcinoma (HCC)	NCT03299946 Single arm Phase 1b	15 patients with locally advanced HCC including patients outside of traditional resection criteria.	Feasibility of neo -cabozantinib and nivolumab.	Primary outcomes, DFS, ORR, OS; Secondary outcomes, AE, pre-op to surgery mPR, CRP.	80% of patients had successful margin negative resection; 42% had major pathologic responses. Enrichment in T effector cells, & TLS, CD138+ PC; TLS found in R. Spatial arrangement of B cells in R only, indicating an orchestrated B cell contribution to antitumor immunity in HCC.
Li K et al., 2020 (98)	Oral	NA	65 pts. with oral cancer.	Oral cancer pts. treated by surgical resection.	Determine gene expression profile & DSF & OS.	Identified up-regulated cytokine & chemokine genes (IL7, LTB & CXCL13) responsible for LN neogenesis correlated with oral cancer-TLS. Improved DFS & OS in patients with higher grades of TLSs. Positive intratumoral (33.8%) and peritumoral (75.4%) TLS detection rates found with intratumoral TLSs significantly associated with decreased P53 & Ki67 scores.
Lutz et al., 2014 (141)	Pancreatic ductal adeno- carcinoma (PDAC)	NCT00727441 Phase 2 Randomized 3-arm vaccine trial	39 PDAC pts. who underwent surgical resection.	GM-CSF secreting allogeneic vaccine (GVAX) +/- IV oral cyclo-phosphamide.	Safety, feasibility, & toxicity. ICB alterations of TME.	Histologic evidence of TLS in 85% of specimens post vs. pre-vac. Improved post-vac responses. Involved in 5 immune-cell activation and trafficking signaling pathways. Suppressed Treg pathway & enhanced Th17 pathway associated with improved survival, enhanced post-vac mesothelin-specific T-cell responses, & increased intratumoral Teff: Treg ratios. Infiltration of T cells & TLS development was in TME. Post-GVAX T-cell infiltration & aggregate formation resulted in upregulation of PD-1-PD-L1 pathway.
Ryan et al., 2020 (170)	Prostate (PCa)	NCT01804712 Pilot/Phase 1, open label, single arm	8 high risk PCa patients.	Neo anti-CD-20 immuno- therapy with Rituximab.	Primary outcome histological ORR after 1 C; secondary, PSA, PB B cell, CXCL13 serum levels.	Mean CD20 density in tumor of treated group significantly lower than control. Mean CD3 density in tumors was significantly decreased in treated group. Neo rituximab was well-tolerated & decreased B- & T-cell density within high-risk PCa tumors. CD20, CD3 & PD-L1 staining primarily occurred in TLS. Rituximab reduced tumor infiltrating B & T-cell density in TLSs denoting inter-dependence between PCa B & T cells.
Helmink et al., 2020 (110)	Melanoma	NCT02519322 Phase 2	Patients with stage IIIB-IV melanoma.	Neo nivolumab (anti-PD-1) +/- ipi (anti-CTLA-4) or relatimab (anti-LAG-3).	Primary outcome, PR. Secondary, OR immuno-logical response, RR. AE, Biomarker & 3 MICs.	Higher RR in tumors enriched with higher expression of B cell-related genes. MIC with the highest expression of B cells exhibited higher OS. CD20+ B cells localized in TLS of R & promoted T-cell activity in the TME.
Cottrell et al., 2018 (171)	Non-small cell lung cancer (NSCLC)	NCT02259621 Phase 2	20 Pts with stage I-IIIA NSCLC.	Neo nivo.	Safety, feasibility, AE, radiographic response	Two (10%) had a pCR, seven (35%) had a pPR, and four (20%) had a pNR. Of the total 9 patients with a major pathologic response, 7 (78%) exhibited TLS.
Campbell et al., 2021 (118)	Metastatic renal cell carcinoma (mRCC)	NCT02626130 Pilot	18 patients with CC & 11 patients with non-CC histologies.	anti-PD-1 or nivo + Ipi.	Primary endpoint, safety; secondary endpoints, ORR, PFS, & immune monitoring.	Trem + cryoablation is feasible, modulates the immune microenvironment, & leads to significant increase in immune cell infiltration in CC. TLS is observed in CC, but not non-CC mRCC in cryoablation plus Trem therapy.
Carril-Ajuria et al., 2022 (119)	Metastatic clear cell renal cell carcinoma (mccRCC)	NIVOREN Phase II (Correlative analysis) GETUG-AFU 26 study	44 mccRCC treated with nivolumab.	Trem (anti-CTLA-4) (n = 15) or without (n = 14) cryoablation in patients with mRCC.	Primary outcome, safety; secondary outcome, best overall response, AE, & association of biomarkers, with OS, PFS, and response.	Baseline unswitched memory B cells (NSwM) enriched in R & associated with improved OS and PFS. Tfh cells inversely correlated with IL-6 & CXCL13. BAFF significantly associated with worse OS in discovery & validation cohorts. R were enriched in circulating Tfh cells and TLS. Circulating NSwM B cells positively correlated with Tfh, TLS, and CD20^+^ B cells at the tumor center & inversely correlated with CXCL13 and BAFF.
Petitprez et al., 2020 (112)	Soft tissue sarcoma	NCT02301039 SARC 028 Phase 2	608 tumors across sarcoma tissue types analyzed.	Nivolumab in patients with mccRCC.	MCP-counter was used to establish 5 different SICs.	SICs Identified. Immune low (A,B), immune high (D, E), and highly vascularized (C). High expression of B cell lineage & association with TLS based on the elevated expression of CXCL13 had the highest ORR and PFS. Group E had TLS containing T cells, follicular DCs, and B cells.
Italiano et al., 2022 (113)	Sarcomas	NCT02406781 PEMBRO-SARC Multiple centers Multi-cohort basket trial 6 parallel single-arm Phase 2 trials	240 patient specimens. LMS, UPS, other STS, GIST, osteosarcoma.	Pembro in soft tissue sarcoma.	Primary outcome, efficacy & safety of Pembro & low dose cyclo-phosphamide.	20% had TLS (700 + CD3+/CD20+ cells) & 73% were included. Median follow-up was 15.6 months. The 6-month PFS was 4.9 (TLS-enriched) vs 1.5 months (all-comer population). 6-month NPR was 40% vs. 4.9% favoring TLS population. TLS potential predictive biomarker in advanced STS to improve patients’ selection for pembrolizumab treatment.
Gao et al., 2020 (115)	Urothelial cancers	NCT02812420 Pilot Phase 1 Neo trial	24-Cisplatin-ineligible patients with UC with high-risk features (bulky tumors, LVSI, variant histology, HG disease, and hydro-nephrosis.	Pembro with low-dose cyclo-phosphamide in independent populations.	Primary endpoint, safety, AE, toxicity.	Higher density TLS found in pretreatment tumor specimens of R vs. NR & associated with longer RFS and OS. Pretreatment samples of CD40+ cells had a significantly higher density of B, CD4+, and CD8+ in pretreatment samples. Higher expression of POU2AF1, a gene that defines TLS, and plays a role in GC initiation. pCR achieved in 37.5%.
van Dijk et al., 2020 (116)	Urothelial cancer	NCT03387761 NABUCCO trial Phase 1B feasibility trial	24 patients with stage III UC.	Neo durvalumab (anti-PD-L1) plus Trem (anti-CTLA-4).	Primary endpoint, feasibility to resect within 12 wks., safety, and efficacy.	Eleven (46%) of patients had a pCR. No correlation between TLS quantity & response. Immature TLS formation higher in tumor specimen without a CR. TLS induction observed in R. TLS presence does not predict response to IMTX.
van Dijk et al., 2021 (117)	Urothelial cancer	NCT03387761 NABUCCO trial Phase 1B	24 patients with stage III UC.	Two doses of neo/ipi (anti-CTLA-4) & nivo (anti-PD-1).	MIF analyzed immune cells in tumors +/-pre-op anti-PD1/CTLA-4.	Specific TLS clusters identified based on immune subset densities (CD3, CD8, FoxP3, CD68, CD20, PanCK, DAPI). Tumors not responsive to IMTX enriched for FoxP3+ T-cell-low TLS clusters after treatment. TLS with low MPs significantly higher after pre-op IMTX compared to untreated tumors. Submucosal TLS had more Th cells & enrichment of early TLS than TLS located in deeper tissue & displayed a lower fraction of secondary follicle like TLS than deeper TLS.

AC, Adenocarcinoma; Ag, Antigen; BAFF, B cell activating Factor; CAF, Cancer-associated fibroblasts; CC, clear cell; CBR, clinical benefit or response; CR, complete response; CIN, Cervical Intraepithelial Neoplasia; CTLA-4, cytotoxic-T-lymphocyte-associated protein 4; C, cycle; DFS, disease free survival; FACS, fluorescence-activated cell sorting; GIST, gastrointestinal stromal tumor; GM-CSF, granulocyte-macrophage colony-stimulating factor; GVAX, GM-CSF-secreting, allogeneic PDAC vaccine; HG, high grade; ICB, immune checkpoint blockade; ICI, Immune checkpoint inhibition; IMTX, immunotherapy; IM, intramuscular; ipi, ipilimumab; HTS, High Throughput Sequencing; LAG-3, lymphocyte-activation gene 3; LINE1, Long-interspersed element 1; LMS, leiomyosarcoma; LVSI, lymphovascular space invasion; MPs, macrophages; mccRCC, metastatic clear cell renal cell carcinoma; MCP, microenvironment cell populations; MIC, melanoma immune class; MIF, multiplex immunofluorescence; MMRd, mismatch repair deficient; mRCC, metastatic Renal Cell Carcinoma; Neo, Neo-adjuvant; Nivo, nivolumab; NPR, non-progression rate; NR, non-responders; NSCLC, non-small cell lung cancer; NSMP, no specific molecular profile; NSwM B cells; Baseline unswitched memory B cells; OC, Ovarian cancer; ORR, Overall Response Rate; OS, overall survival; PBMC, Peripheral Blood Mononuclear Cells; pCR, pathologic complete response; PD-1, programmed cell death protein 1; PFS, progression free survival; pNR, pathological non-response; pPR, pathologic partial response; PR, Partial response; Pembro, Pembrolizumab; POLE, DNA polymerase epsilon; Post-vac, Post-vaccination; Pre-vac, pre-vaccination; PC, Prostate cancer; Pts, Patients; R, responders; RFS, recurrence-free survival; RR, Response rate; SD, Stable Disease; SIC, sarcoma immune class; STS, soft tissue sarcoma; TCGA, The Cancer Genome Atlas; T_eff_, effector T-cell; TLLS, Tertiary lymphoid-like structures; TLS, tertiary lymphoid structures; Tfh, T follicular helper; TME, tumor microenvironment; TX, Treatment; T_reg_, regulatory T cell; Trem, tremelimumab; UC, urothelial carcinoma; UPS, undifferentiated pleomorphic sarcoma; wks., weeks. PubMed search through April 2024.

**Table 2:** Tertiary lymphoid structure involvement in ovarian cancer.

Author, Year, [Ref]	Goal/Intervention	Patients/Methods	Relevant Results	TLS Involvement and Conclusions
Kroeger et al., 2016 (172)	Determine colocalization patterns, phenotypes, & gene expression profiles of tumor associated T & B lineage cells in HGSOC.	Multicolor IHC, flow cytometry & bioinformatic analysis of gene expression data from TCGA.	T-and B- cells colocalized in four types of lymphoid aggregate, ranging from small, diffuse clusters to large, well-organized TLS resembling activated LN. PCs associated with the highest levels of CD8+, CD4+ & CD20+ TIL, & numerous cytotoxicity-related gene products. CD8+ TIL carried prognostic benefit only in the presence of PCs & these other TIL subsets. PCs were independent of mutation load, BRCA1/2 status, & differentiation Ags but positively associated with CTA.	B and T cells co-localize in 4 lymphoid patterns, including large, well- organized TLS. TLS frequently surrounded by dense infiltrates of PC, comprising up to 90% of tumor stroma. Tumor infiltrating-PC expressed mature, oligoclonal IgG transcripts indicative of Ag-specific responses & are associated with TLS, cytolytic T-cell responses, & superior prognosis in OC.
Yang et al., 2021 (124)	Determine therapeutic effect of CXCL13 & PD-1 blockade.	264 HGSOC patients/2 cohorts & 340 HGSOC TCGA cases used. Survival compared in pt. subsets (Kaplan-Meier analysis). Therapeutic effect of CXCL13 & PD-1 blockade, validated in murine models & human HGSOC tumors. Spatial correlation between CXCL13, CXCR5, CD8, & CD20 evaluated by IHC & IF.	CXCL13 associated with CD20+ B cells predicted better patient survival. Combination of CD8+ T cells, CXCL13, & CXCR5 was an independent predictor for survival. Tumors with high CXCL13 expression had increased infiltration of activated and CXCR5 expressing CD8+ T cells. Murine studies: CXCL13 & anti-PD1 therapy showed a CD8+ T cell dependent retarded tumor growth with increased infiltration of cytotoxic CD8+ T cells & CXCR5-CD8+ T cells.	CXCL13 colocalizing with TLSs shapes anti-tumor microenvironment by maintenance of CXCR5+CD8+ T cells in TLS. TLS prognostic benefit is only relevant in the presence of CXCL13. High CXCL13 expression associated with prolonged survival. Supports a new CXCL13 & PD-1 blockade clinical trial in HGSOC.
Chen S, 2022 (122) NCT02901899	Determine if hypomethylating agent guadecitabine in a phase II study improves ICI in platinum-resistant OC.	35 platinum-resistant OC patients (normal organ function, measurable disease, up to 5 prior treatments). Primary endpoint: ORR. Secondary endpoints: CBR, PFS, & Toxicity.	3 patients had PR, 8 had SD, CBR of 31.4%. Median duration of clinical benefit was 6.8 mo. Anti-tumor immunity activated in post-treatment biopsies as seen with methylomic & transcriptomic analyses. PBMCs showed higher frequency of naive &/or central memory CD4+ T cells and classical monocytes in patients with a durable CBR.	A higher baseline density of CD8+ T cells & CD20+ B cells & the presence of TLS in tumors were associated with a durable CBR.
Gaulin, 2022 (22) NCT02853318 (21)	Determine factors contributing to durable response in R.	40 advanced OC patients with previous multiple lines of therapy treated with Pembro, Beva, and Cytoxan therapy (21). Improved PFS & OS.	Transcriptomic assessment indicative of a more favorable immune signature with B & T cells, CD40, Ag presenting, cytokine, & TLS, at baseline & during treatment. Immunoproteins are upregulated intratumorally. Immune cells move from stroma into the TME.	Multi-omics assessment of R and NR pt. samples indicative of cellular and humoral response, including TLS signature, in R who have a durable response to Pembro, Beva, and Cytoxan therapy.
Ozmadenci et al., PNAS 2022 (148)	Determined the effects of tumor intrinsic genetic or oral small molecule FAK inhibitor (FAKi; VS-4718) *in vivo*.	Used the Kras, Myc, FAK (KMF) syngeneic ovarian tumor mouse model containing spontaneous FAK/PTK2 gene gains.	Blocking FAK activity decreased tumor burden, suppressed ascites KMF-associated CD155/PVR levels, & increased peritoneal TILs.	FAKi + 1B4 TIGIT blocking antibody maintained elevated TIL levels, reduced TIGIT+ T reg cell levels, prolonged host survival, increased CXCL13 levels, & led to the formation of omental TLS. These results support FAK & TIGIT targeting as a rational immunotherapy combination for HGSOC.
Ukita, M et al., 2022 (79)	Determine TLS distribution in TME in relation to TILs & related gene expression in HGSOC specimens.	Used OC cases registered in TCGA. TCGA-RNA seq used, & KOV microarray data.	CXCL13 gene expression correlated T & B cells infiltration. CXCL13 was a favorable prognostic factor. CD8+ T cells and B cell lineages in TME significantly improved the prognosis of HGSOC & correlated with presence of TLS. CXCL13 expression coincident with CD4+ T cells in TLS & CD8+ T cells in TILs & shifted from CD4+ T cells to CD21+ follicular DCs as TLS matured.	CXCL13 gene expression correlated with TLS presence. CXCL13-producing CD4+ T cells are involved in the early stage of TLS formation. TLS formation was associated with CXCL13-producing CD4+ T cells & TLS facilitated the coordinated anti-tumor response of cellular & humoral immunity in OC.
Hou, Y et al., 2023 (104)	Determine related gene signature of TLS in OC.	TLS gene signature constructed in TCGA dataset & validated in the GSE140082 dataset.	High expression of gene signatures positively correlated with developed immune infiltration & reduced immune escape. Quantified TILs (CD20+ B cells & CD8+ T cells) in OC patients. PD-L1 proved predictive value of immunotherapy for gene signature. Signature showed a better correlation between TMB & classical checkpoint genes.	OC TLS gene signature (CETP, CCR7, SELL, LAMP3, CCL19, CXCL9, CXCL10, CXCL11, CXCL13) identified & validated. Signature predicts prognosis & immunotherapy benefit. Confirmed improved survival values of TLS & TLS play an important role in tumor immunity. Gene signature proposed as prognostic biomarker & means to guide immunotherapy in OC.
Zhang K., et al., 2023 (71)	Determine presence of TLSs, their potential clinical significance & association with TME, & immunotherapy response in HGSOC.	HGSOC TCGA cohort of 376 pts with RNA seq data, 74 with H&E; 212 with microarray data (GEO cohort). TLS pathological sections with TLS number assessed by 12-chemokine transcriptomic signature.	TLSs located mainly in stroma & invasive margin of tumor (H&E). HGSOC patients are divided into a low-TLS & high-TLS cluster. Expression of 12 chemokines significantly higher in samples abundant with TLSs.	TLS associated with favorable response to ICI & are a favorable independent prognostic factor in HGSOC. High-TLS cluster had better clinical prognosis, higher immune infiltration and TMB score, more biological pathways, & higher immune checkpoint expression. TLSs strongly correlated with the immune-responsive microenvironment.
Lu, H et al., 2023 (105)	Determine if tumor & local lymphoid tissue interaction decide prognosis in HCSOC.	Immunogenomic analysis of 242 HGSOC cases.	TLS associated with B & T cell activation in OC & predict survival. TLS loss/dysfunction linked to chromosome 4q deletion/DCAF15 amplification.	Presence of TLS in HGSOC tumors associated with B cell maturation & cytotoxic tumor-specific T cell activation & proliferation. Copy-number loss of IL15 & CXCL10 may limit TLS formation in HGSOC.
Feng et al., 2023 (147)	Determine the mechanism cdk4/6i promotes TLS formation & if TLS affect OC prognosis.	Mouse model & HTS used to explore potential mechanisms.	After CDK4/6i treatment, TLS observed & associated with favorable OC prognosis. CDK4/6i promoted TLS formation, enhanced immunotherapeutic effect of anti-PD-1, & may be modulated through SCD1, ATF3, & CCL4.	TLS associated with favorable OC prognosis following CDK4/6i. CDK4/6i may be a therapeutic option for OC, alone & in combination with anti-PD-1 therapy.
Kasilova et al., 2024 (72)	Determine distinct immune aggregate patterns, organization, & maturity associated with HGSOC.	FFPE OC & LC cohorts, IHC, flow, & RNAseq.	mTLS formed in limited HGSOC with high TMB & are associated with increased intratumoral density of CD8+ effector T cells. An ICI-resistant TIM3+PD1+ phenotype is more prevalent in HGSOC & supported by less mTLS.	TLS and B cells determine clinically relevant T cell phenotypes in OC. OC associated with low density of Tfh cells, low mTLS that might not preserve an ICI-sensitive TCF1+PD1+ CD8+ T cell phenotype.
Xu et al., 2024 (73)	Determine how spatial heterogeneity contributes to HGSOC progression & early relapse.	Profiled an HGSOC tissue microarray of 42 patients matched longitudinally.	Spatial patterns associated with early relapses (Changes in T cell location, malformed TLS-like aggregates, & increased podoplanin-positive CAFs). PC distribute to 2 different compartments associated with TLS-like aggregates & CAFs.	Poorly formed TLS-like aggregates play a role in early relapses (<15 mo.) of HGSOC patients. TLS-like aggregates appear to associate with PCs in compartments with distinct microenvironments.
Lanickova et al., 2024 (168)	Determine if neoadjuvant chemotherapy paclitaxel-carboplatin had impact on immunological configuration of paired primary and metastatic HGSOC biopsies.	Used transcriptomic, spatial, and functional assays.	TLS maturation is associated with increased intratumoral density of ICI sensitive TCF+PD1+CD8+T cells. Chemotherapy + PD-1 targeting ICI provides a survival benefit. Increase in effector CD8+ T (Teff) cells, B cells, DC, and TLS-associated cells, such as follicular helper T cells, which collectively contribute to cancer cell killing also observed.	Neoadjuvant chemotherapy promotes TLS formation, adaptive immunity, and maturation in metastatic HCSOC lesions. Intracellular calreticulin (CALR) expression within cancer cells, a marker of endoplasmic reticulum stress and potential cell death, was increased. Suggests clinical trial with chemo + ICI.
Zhang L et al., 2024 (173)	Determine antibody-secreting B lymphocytes from OC patients.	Profiled stably maintained cell lines with flow, and B cell receptor sequencing. Tumor samples used for spatial profiling with chip cytometry.	Presence of EBV proteins Original tumors had high frequency of tumor infiltrating B cells present as lymphoid aggregates or TLS.	Antigens recognized coil-coil domain containing protein 155 (CCDC155), growth factor receptor-bound protein 2 (GRB2), and pyruvate dehydrogenase phosphatase 2 (PDP2).
MacFawn et al., 2024 (125)	Determine TLS in various locations of OC development.	Spatial analytes used. Spatial transcriptomics Digital Spatial Profiling.	Pro-tumorigenic stroma could limit TLS formation. Cancer-educated Mesenchymal Stem Cells (CA-MSC) could decrease antitumor efficacy.	TLS less developed in tumors originating in ovary tumor vs FT or OM. Immune cell activity increases when residing in more developed TLS and produces a prognostic, spatially derived signature from HGSOC tumors. CA-MSC may contribute to more suppressive TME in ovary limiting TLS, T and B cell and antibody infiltration, and overall decreased anti-tumor activity.
Rosario et al., 2024 (23) NCT02853318 (21)	Multi-omics assessment of durable clinical benefit (DCB) vs. limited clinical benefit (LCB) patients.	40 pts accrued. Bulk RNA seq, metabolomics, microbiome, immune studies, and DSP performed.	Infiltration of immune cells from stroma to tumor. Coordinated increase and movement of T and B cell infiltrates. Additional immune cell signatures associated with CD40 Ag, Ag presentation, cytokine presence, and indications of TLS	Identification of Tumor-Immune-Gut Axis. Presence of TLS signatures and humoral and cellular involvement of TLS, humoral and cellular anti-tumor responses, and metabolomic pathways in DCB vs. LCB patients.
Westbom-Fremer et al., 2025 (174)	Investigated mTLS, iTLS & LA in PTs & pMets of HGSC.	Whole H&E slides interrogated for mTLS and LA in a cohort of 130 cases with stage III-IV HGSC. Immune cell tumor infiltration evaluated using TMA on cases with single chromogenic IHC. MIF further performed on select PT and pMet samples.	mTLS more common in pMets than in PTs but did not have an independent prognostic impact on overall or PFS. mTLS presence correlated with intratumoral infiltration of CD8+ cytotoxic T cells, FOXP3+ Tregs and PD-1+ lymphocytes in pMets only. mTLS cell composition similar between PTs and pMets, but outer zones of mTLS in PTs were more immune cell rich. No iTLS identified.	Differences in TLS presence and cellular elements between PTs and synchronous pMets important for understanding mechanisms of immune evasion and initiation of tumor targeted immunity. Could not deduce an independent prognostic impact of mTLS and LA in this case cohort. Suggests anatomical site relevant for modulation of immune landscape especially in recurrent setting.

Ag, Antigen; ATF3, Activating transcription factor 3; Beva, Bevacizumab; CCL4, chemokine ligand 4; Cdk4/6i, Cyclin dependent kinase 4 and 6 inhibitor; CAF, Cancer-associated fibroblasts; CBR, clinical benefit rate/response; CR, complete response; CTA, Cancer testis antigen; DC, Dendritic Cells; FAK, Focal adhesion kinase; FAKi, Focal adhesion kinase inhibitor; FFPE, formalin fixed paraffin embedded; FT, Fallopian Tube; H&E, Hematoxylin and Eosin; HGSC, High grade serous cancer; HGSOC, High grade serous ovarian cancer; HTS, High throughput screens; ICI, Immune checkpoint inhibition; IF, Immunofluorescence; IgG, Immunoglobulin G; IHC, Immunohistochemistry; iTLS, immature Tertiary lymphoid structures; KMF, Kras, Myc, FAK syngeneic mouse; LN, Lymph node; LA, Lymphoid aggregates; LC, Lung cancer; MIF, multiplex immunofluorescence; mTLS, mature tertiary lymphoid structure; NR, non-responders; OC, Ovarian cancer; OM, Omentum; ORR, Overall Response Rate; OS, overall survival; PBMC, Peripheral Blood Mononuclear Cells; PD-1, programmed cell death protein 1; PD-L1, Programmed death-ligand 1; PFS, progression free survival; PR, Partial response; Pembro, Pembrolizumab; PTs, primary adnexal tumors; pMets, omental/peritoneal metastases; PVR, Polio virus receptor; R, responders; SCD1, Stearoyl-CoA desaturase 1; SD, Stable Disease; TCGA, The Cancer Genome Atlas; TIGIT, T cell immunoglobulin and ITIM domain; TMA, tissue microarray; TLS, tertiary lymphoid structures; Tfh, T follicular helper cell; TME, tumor microenvironment; TIL, Tumor infiltrating lymphocyte; T_reg_, regulatory T cell; TMB, Tumor mutational burden; wks., weeks. PubMed search (tertiary lymphoid structures and ovarian and cancer) through January 2025.

Pembrolizumab monotherapy was assessed in soft tissue sarcoma where one out of 5 sarcoma immune classes were characterized by high immune, TLS, and B cell lineage expression which significantly improved OS and overall response rate (ORR) (112). Sarcoma patients treated with pembrolizumab with low-dose cyclophosphamide had three times greater PFS in the TLS-enriched populations vs. all-comers (113), while NSCLC tumors from patients treated with either the PD-L1 inhibitor atezolizumab or chemotherapy showed B cells associated with extended OS following PD-L1 blockade. B cells and PC were also associated with TLS and organized lymphoid aggregates, with increased PC signatures predictive of OS in patients treated with atezolizumab only (114).

Combination immunotherapy trials have shown improved outcomes in some solid tumor cancers. Neoadjuvant anti-PD-L1 (durvalumab) plus anti-CTLA-4 (tremelimumab) treatment of cisplatin-ineligible patients with urothelial cancer (UC) had higher density TLS in pretreatment tumor specimens of responders (R) associated with longer recurrence-free survival (RFS) and OS (115). In previous neoadjuvant ipilimumab (anti-CTLA-4) and nivolumab (anti-PD-1) studies prior to surgical resection, while no correlation was observed between TLS quantity, TLS induction was seen in R, with immature TLSs higher in patient tumor specimens that did not have a complete response (CR) (116). Enriched FoxP3^+^ T cell-low TLS clusters were observed after anti-PD-1/CTLA-4 immunotherapy in unresponsive tumors in another study, while submucosal TLS had more pronounced T-helper cells, enrichment of early TLS, and displayed a lower fraction of secondary follicle-like TLS than TLS located in deeper tissue (117).

Other combination therapy, including immunotherapy, had varied results in smaller trials. Cryoablation plus tremelimumab modulated the immune microenvironment with increased immune cell infiltration in patients with clear cell (CC) metastatic renal cell carcinoma (mRCC); however, TLS were only observed in CC mRCC patients and not non-CC (118). An enhanced immune response was observed in R in a second trial where CC mRCC patients treated with nivolumab were enriched in circulating Tfh cells, TLS, and baseline NSwM B cells, and were also associated with improved OS and PFS (119). In a small single arm phase 1b study in 15 HCC patients testing neoadjuvant cabozantinib, a tyrosine kinases inhibitor, and nivolumab, TLS were found in R, with an enrichment in T effector cells, TLS, and CD138^+^ PC. Here, a distinctive spatial arrangement of B cells contributed to antitumor immunity in R vs. Non-responders (NR) (120).

### Tertiary lymphoid structures in ovarian cancer

Several studies, including data collected through The Cancer Genome Atlas (TCGA), confirm a connection between TLS, B and T cells, and OC (**Table 2**). Increased patient survival was observed between tumor-infiltrating B cells and CD8^+^ T cells, offering possible mechanisms for B cell involvement in cellular immunity, including secreting polarized cytokines, serving as APCs, or organizing centers for TLS (121). B cells colocalized to large, well-organized TLS, while dense infiltrates of PC surrounded TLS, which are associated with the highest levels of CD8^+^, CD4^+^, and CD20^+^ TIL, as well as cytotoxicity-related gene products. TLS also correlated with CXCL13 gene expression, infiltration of T cells and B cells, and improved prognosis for HGSOC (79). The coexistence of CD8^+^ T cells and B cell lineages in the TME significantly improved the prognosis of HGSOC and correlated with the presence of TLS. CXCL13 expression predominantly coincided with CD4^+^ and CD8^+^ T cells in TILs, shifting from CD4^+^ T cells to CD21^+^ FDCs as TLS matured.

A phase II clinical trial with the hypomethylating agent guadecitabine and the anti-PD1 inhibitor pembrolizumab resulted in a clinical benefit rate (CBR) of 31.4% (8.6% partial responders and 22.9% stable disease) (122). Naive and/or central memory CD4^+^ T cells and classical monocytes were observed at a higher frequency in patients with a durable CBR, while TLS present in tumors were associated with a durable CBR and higher baseline density of CD8^+^ T cells and CD20^+^ B cells. Updated efficacy and OS survival data for an open-label single-arm nonrandomized phase 2 trial (NCT02853318) by Zsiros et al. (21) was presented in 2022 (22) for a triple combination therapy (pembrolizumab, bevacizumab, and oral metronomic cyclophosphamide) conducted in a heavily pre-treated OC patient population. Originally, an ORR of 47.5% was obtained, along with a median PFS of 10 months, which was over 4 times better than ORR (8%) or PFS (2.1 mo.) in single-agent ICI, and better than bevacizumab and cyclophosphamide combination therapy (25). Thirty percent of patients had disease control, with 95% having a clinical benefit, with limited toxicity and good quality of life (QoL) reported. Some exceptional R survived more than three years, and one up to five, with multi-omics performed on collected biospecimens from this study (23) suggesting involvement of TLS, humoral and cellular anti-tumor responses, and metabolomic pathways in DCB vs. LCB patients. These results contributed to changes in NCCN guidelines (123).

Additionally, in studies to predict immunotherapy response to PD-1 blockade, HGSOC samples from two patient cohorts and an HGSOC cohort from TCGA were analyzed to assess the relationship between TME and follicular cytotoxic CXCR5-CD8^+^ T cells. Again, high expression of CXCL13 was associated with prolonged survival, and, combined with CXCR5 and CD8^+^ cells, was an independent predictor for survival. CXCL13 also carried prognostic benefit, but only with TLS, and was also associated with CD20^+^ B cell clusters, predicting better patient survival when both were present (124). Along with the 9-gene and 12-gene CK signatures, the combinations of immune cell markers, as well as CXCL13, are the strongest biomarker front runners for prolonged survival in combination with mTLS.

**Figure 2:**
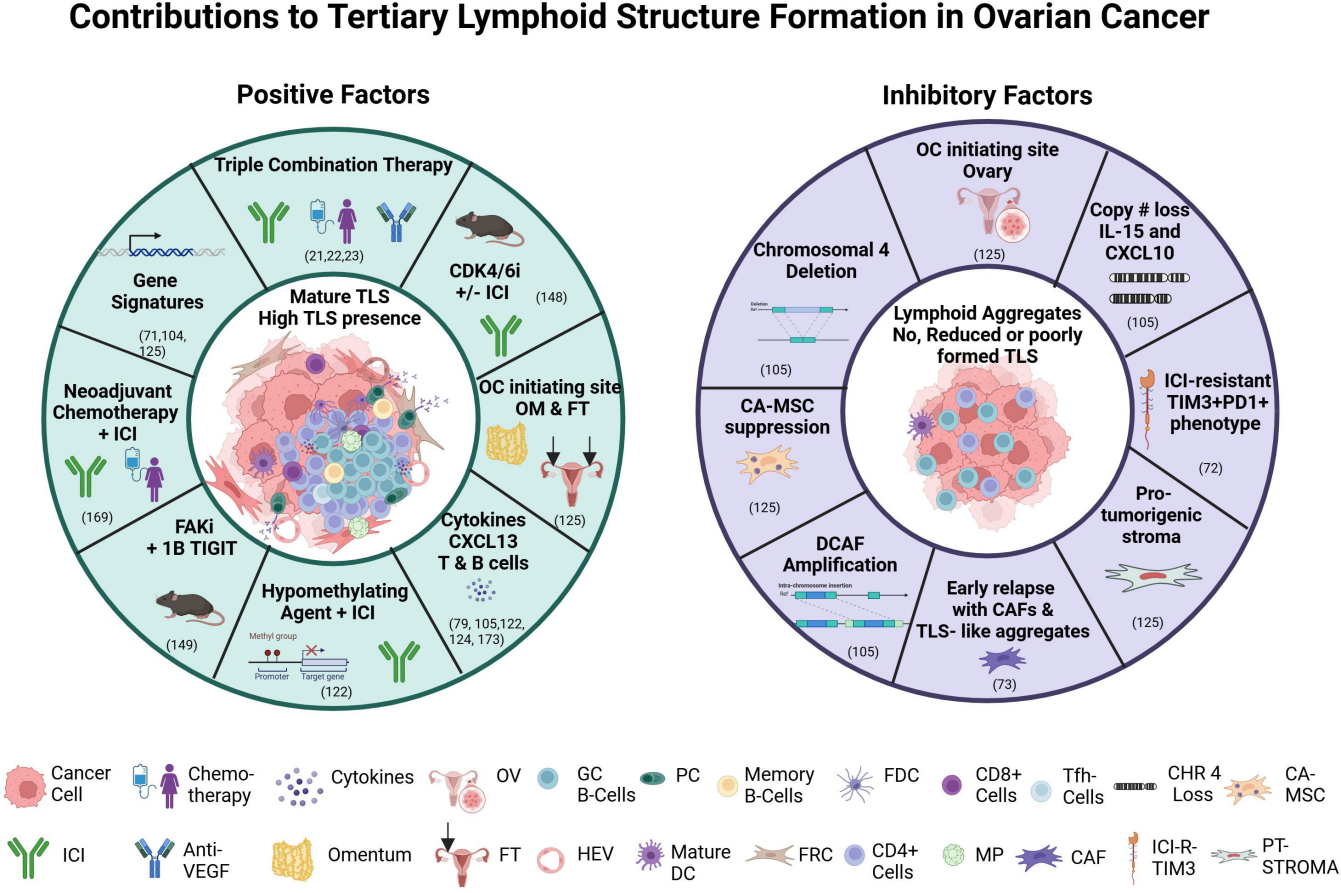
Factors contributing to or inhibiting tertiary lymphoid structure formation. Abbreviations: anti-PD-L1, Programmed death ligand 1; CDK 4/6i, cyclin dependent kinase 4/6 inhibitor; CAF, cancer associated fibroblasts; CA-MSC, cancer-educated mesenchymal stem cell; CHR 4, chromosome 4 loss; DC, dendritic cell; FAKi, focal adhesion kinase inhibitor; FDC, follicular dendritic cell; FRC, fibroblastic reticular cell; FT, fallopian tube; GC B-cells, germinal center B cells; HEV, high endothelial venule; ICI, immune checkpoint inhibitor; ICI-R-TIM3, ICI-Resistant TIM3; MP, macrophage; OM, omentum; OC, ovarian cancer; OV, ovary; PC, plasma cell; PT-STOMA, pro-tumorigenic stroma; TIGITi, T cell immunoreceptor with Ig and ITIM domain inhibitor; TIM3, T cell immunoglobulin and mucin domain 3;TLS, tertiary lymphoid structures; VEGF, vascular endothelial growth factor. Figure generated with BioRender.com.

Recently, MacFawn et al. (125), using spatial transcriptomics and multiplex immunofluorescence, demonstrated TLS in HGSOC differ significantly by anatomical site resulting in altered TLS activity and patient prognosis. Composition, function, activity, and number of TLS are distinct between OC originating in the ovary (OV) compared to the fallopian tube (FT) and omentum (OM) and this directly impacts TLS activity, immune function, and survival. Ovarian tumors originating from the FT and OM had more TLS that are more functionally active compared to OV, which are less mature and fewer in number, as well as have a reduced B to T cell ratio, fewer HEVs, and less GC, thus reduced antitumor immunity. Not only were B cells markedly reduced in OV compared to OM or FT, but there are decreased markers (MZB1+) of antibody production in OV. The TLS 12 CK-gene signature associated with improved survival was observed in FT and OM but indicated poor survival in OV. Another major finding was the impact of stromal cells in the OV TME (126). Cancer educated mesenchymal stem cells (CA-MSC) have been identified in the OV, where the CA-MSC signature appears to override the benefit of the TLS signature (125). These cells previously have been associated with reduced survival and metastasis (127). mTLS differences were also observed in adnexal tumors (PTs) and synchronous omental/peritoneal metastases (pMets) in HGSOC. pMets had more mTLS and immune components (CD8+ cytotoxic T cells, FOXP3+ Tregs and PD-1+ lymphocytes) than PT, but an independent prognostic impact of mTLS could not be obtained. A more immune cell rich outer zone of mTLS was observed in PTs, but not pMets. This suggests the anatomical site, as well as deciphering what is involved in immune response and evasion, are relevant when considering what signals to modulate to improve the antitumor response. TLS have also been assessed in HGSOC progression & early relapses using spatial heterogeneity. Spatial patterns associated with early relapses included changes in T cell location, TLS-like aggregates which appear malformed, and an increased presence of PDPN-CAFs. These poorly formed TLS aggregates are potential indicators of early relapse in HGSOC (73). **Figure 2** summarizes factors that impact TLS in OC and identifies potential pathways or strategies that may be manipulated or explored that may increase TLS formation and improve therapeutic outcomes.

While this review has predominantly focused on the role of TLS in the context of ICI, additional immunotherapeutic approaches and treatment strategies are in development for OC where TLS are likely to also play a functional role. Given that OC is readily infiltrated by T and NK cells, leveraging these cell subsets may represent particularly attractive therapeutic opportunities. This includes adoptive T cell therapies (ACT) or T cell activating modalities, such as the delivery of expanded TILs, or vaccine-primed T cells, infusion of engineered TCR-T or CAR-T cell therapies, as well as treatment with BiTEs to redirect T cells for tumor targeting (128, 129). A major focus of ACT-based modalities hinges on successful target identification, which includes in OC MUC 16, FRα, B7H3, mesothelin, and TAG72 (130), to name a few, as well as strategies to overcome inherent limitations of antitumor T cell responses, including lack of T cell persistence, tumor heterogeneity, loss of functionality, and impaired trafficking towards the TME, all of which may benefit from the presence of TLS. A recent publication from our group addressed some of these barriers to magnify therapeutic responses by using BiTE-secreting T cells targeting FRα present on most OC cells. Engineered T cells secreting FR-alpha engagers (FR-B T cells) not only effectively killed targeted OC cells but also redirected endogenous host T-cells to kill tumor cells (131). Follow up studies are currently underway to understand how TLS may influence the local organization of both transferred FR-B T cells and endogenous immune cells in the TME in orchestrating the antitumor response.

Other ACT strategies include using NK cells, which can kill cancer cells without prior sensitization and are not antigen dependent. Several studies have been conducted using murine models (132–134) to explore improving cytotoxicity and longevity of NK cells. One example, addition of cytokines IL-2, IL-15, and IL-18, promotes activation and proliferation of NK cells, as well as secretion of INF gamma and inhibition of OC cells (135). This strategy generated cytokine-induced memory-like NK cells. Additional IL-15 or IL-15 super agonist complexes (N-803 or ALT-803) also promoted proliferation of NK cells with additional secreted factors including TNFα, CXCL10, and CD107a, which may enhance the function of NK cells (132, 136, 137). Only a limited number of OC patients have been treated clinically with NK ACT (138, 139), with some patients achieving stable disease with mild side effects (139). Several pre-clinical NK studies propose strategies to maximize effects of NK ACT including genetic modifications, combination therapy with ICI, targeted therapies, cytokines, and CAR-NK constructs, which currently are in early phase clinical trials (140)

## Therapeutic strategies to induce TLS

Because TLS appears to play a major role in the anti-tumor response, identifying ways to induce TLS appears to be an important strategy. Vaccination contributes to induction of TLS and T cell infiltration as evidenced in pancreatic and cervical cancer. For example, in pancreatic adenocarcinoma patients with GM-CSF secreting allogeneic vaccine (GVAX) +/- a single IV dose of daily oral cyclophosphamide, eighty-five percent of post-vaccination tumor specimens contained histologic evidence of TLS formation not observed pre-vaccination. T cell infiltration and TLS development occurred in TME, with five signaling pathways involved in regulating immune-cell activation and trafficking associated with improved postvaccination responses differentially expressed, including genes encoding integrins, chemokines/chemokine receptors, and members of the ubiquitin-proteasome system and NK-kappa B pathway (141). In cervical cancer patients, intramuscular injection of a vaccine against HPV16E6/E7 induced TLS (142). This suggests that TLS formation may be needed for optimal vaccine response, since cancer vaccines often have limited efficacy, but also vaccination may be a strategy to induce TLS formation.

Oncolytic virotherapy has been proposed to induce TLS especially with immune checkpoint blockade (ICB) to amplify antitumor immune responses to patients with cancer (143). Because many tumors do not form functional TLS structures, “designer LN” have been proposed via injection of genetically modified immune cells in biomaterial scaffolds (65). The success of bioengineered lymph structures may depend on cellular properties for effective migration, hydrostatic fluid pressures for regulation of lymph flow and extravasation, as well as safety for cancer patients (144).

### TLS in murine models

Several murine models suggest key players and potential strategies for the induction of TLS or new therapeutic strategies +/- immunotherapy. He et al., 2022 (145) have shown an oncolytic adenovirus carrying mIL-15 effectively facilitated activation and infiltration of DC, T, and NK cells into the TME, and induced TLS and vascular normalization. This mechanism appears to be through induced activation of the STING-TBK1-IRF3 pathway in DCs, and not traditional pathways of TLS induction. IKZF1, a gene related to the development of OC, melanoma, breast, and liver cancer, has been proposed to be a key driver of the formation of immature TLS (146). Using CRISPR/Cas9 gene editing to generate novel ID8 derivatives that harbor single and double suppressor gene mutations (Trp53^-/-^ or Trp53^-/-^; Brca2^-/-^) in HGSOC, slower orthotopic tumor growth was observed in the double mutant compared to Trp53^-/-^, with rich intra-tumoral TLS in CD3^+^ T cells (146). TGF-Beta mediated silencing of genomic organizer SATB1 promoted Tfh cell differentiation and formation of intra-tumoral TLS, providing additional insight into how TLS are generated within tumors (82). Accumulated tumor antigen-specific LIGHT^+^CXCL13^+^IL-21^+^ Tfh cells and TLS decreased tumor growth in a CD4^+^ T cell and CXCL13-dependent manner (82).

Finally, two murine studies that are TLS-driven may offer new therapeutic strategies for OC patients. Cdk4/6 inhibitors (CDK4/6i) promoted TLS formation and enhanced the immunotherapeutic effect of anti-PD-1 when used in combination. TLS were associated with a favorable prognosis following CDK4/6i treatment, which may provide a therapeutic option for OC patients when used either alone, or in combination with anti-PD-1 therapy (147). A second study used an oral, small molecule focal adhesion kinase (FAK) inhibitor, FAKi (VS-4718), alone, and in combination with a blocking antibody to the checkpoint protein T cell immunoglobulin and ITIM domain (TIGIT), an inhibitory receptor expressed on lymphocytes that interacts with CD155 expressed on APC or tumor cells to down-regulate T cell and NK cell functions. Using a Kras, Myc, FAK (KMF) syngeneic ovarian tumor mouse model, decreased tumor burden, suppressed ascites KMF-associated CD155/Polio Virus Receptor (PVR) levels, and increased peritoneal TILs were observed with blocked FAK activity. FAKi combined with the 1B4 TIGIT blocking antibody maintained elevated TIL levels, reduced TIGIT^+^ T reg cell levels, prolonged host survival, increased CXCL13 levels, and led to omental TLS formation. These results provide a rationale, especially considering different anatomical sites of OC origin playing a role in humoral and cellular immunity, conducting a FAKi/TIGIT blocking antibody pilot in HGSOC patients (148).

### Influence of the gut microbiome on TLS formation and generation of antitumor immunity

Another inducer of TLS is the gut microbiome, which appears to play a role in modulating the host response to ICI. The microbiome, comprised of trillions of microorganisms lives in a symbiotic relationship with human hosts (149), and actively regulates aspects of host physiology, including immunity (150). The human gut microbiome appears to be associated with TLS, B cells, and the antitumor response (151–153), although the exact mechanism still needs to be elucidated. Advances in identifying potential important bacteria in the microbiome are due to culture-independent genomic sequencing (e.g., 16S rRNA sequencing) at the species and strain level (154). Recently, *Helicobacter hepaticus*, a single immunogenic commensal bacterium, controlled growth in a carcinogen-induced orthotopic CRC mouse model, challenging the premise multiple immune-potentiating microbes are required to shift the host immune system towards potent antitumor immune responses. Generated bacteria-specific CD4^+^ Tfh cells stimulated TLS formation within the tumor and surrounding areas, leading to increased tumor immune infiltration and reduced tumor burden (155). This manipulation at a single taxa level was sufficient to generate robust anti-tumor immunity and TLS formation. TLS induction has also been found in a retrospective study of 60 HCC cases which showed that in an intratumoral TLS group, Lachnoclostridium, Hungatella, Blautia, Gusobacterium, and Clostridium were increased (156).

The gut microbiome is also associated with clinical responses to anti-PD-1 immunotherapy in a variety of cancers (155, 157–163), however, no common overall microbial signal or metabolomic profile has been identified, even within cancers, indicating additional factors must be at play. Higher alpha diversity associated with longer PFS (151) (157) (162), and abundance of specific species has been observed. In melanoma, higher microbial community richness is associated with longer PFS, and abundance of specific Bacteroides species (Ruminococcus gnavus), while the Blautia producta strain is related to shorter PFS (162). PFS also correlated to metatranscriptomic expression of risk-associated pathways of L-rhamnose degradation, guanosine nucleotide biosynthesis, and B vitamin biosynthesis. Higher relative abundance of Ruminococcaceae family of bacteria were also found in melanoma R, as well as anabolic pathway enrichment and enhanced systemic and antitumor immunity, mediated by increased antigen presentation and improved effector T-cell function in the periphery and TME (151).

In two NSCLC studies, patients with high microbiome diversity at baseline (and throughout), had significantly prolonged PFS compared to those with low diversity, and also had a greater frequency of unique memory CD8^+^ T cell and NK cell subsets in the periphery, suggesting a strong association between gut microbiome diversity and specific immune-related systemic populations (157). In clinical response to anti-PD-1/PD-L1 in advanced-stage gastrointestinal (GI) cancer, an elevated Prevotella/Bacteroides ratio was observed in R. A differential abundance of pathways related to nucleoside and nucleotide biosynthesis, lipid biosynthesis, sugar metabolism, and fermentation to short-chain fatty acids (SCFA) was identified in patients’ samples exhibiting differential responses. Gut bacteria associated with SCFA production, including Eubacterium, Lactobacillus, and Streptococcus, were positively associated with anti-PD-1/PD-L1 response across different GI cancer types (161).

The gut microbiome of durable R was enriched for *Akkermansia muciniphila* and *Ruminococcaceae* species in HCC patients treated with anti-PD-1 therapy. In hepatobiliary cancers, seventy-four specific taxa, including Lachnospiraceae bacterium-GAM79 and Alistipes sp Marseille-P5997, were significantly enriched in the CBR group, which achieved longer PFS and OS than patients with lower bacterial abundance (158) and the CBR group was associated with energy metabolism. These bacterial, metabolomic, and immune signatures are potential biomarkers to explore in OC patients treated with immunotherapy. Recently, specific bacteria were identified, including Intestinimonas butyriciproducens and Anaerotignum propionicum (Clostridium propionicum), that were highly abundant both before and after treatment for OC patients that achieved DCB following a triple combination therapy (23). These butyrate-producing bacteria have previously been associated with an enhanced response to immunotherapy (164), and increases of these bacteria in the triple combination therapy were also linked to increased immune and TLS changes in the TME. These results may generate signatures for R and NR of specific combination therapies.

While such findings are encouraging, additional studies, including an improved understanding of the microbiome and metabolic pathways, are needed to delineate nuances of a patient’s likelihood to respond to ICI therapy (163), as well as to drive TLS formation. Opportunities exist for immunomodulation through dietary and gut microbial interventions (165). Manipulating the presence of different bacterial species or metabolic pathways/metabolites, with interventions such as fecal transplants, probiotic use, microbial antigens or proteins, and selective antibiotics, may generate TLS and more favorable patient outcomes using immunotherapeutic approaches.

## Conclusion and future directions

Understanding the complex roles of TLS within the TME is vital for improving therapeutic outcome for cancer patients, especially for those with limited treatment options at late-stage disease, as is the case with OC patients. Unlike SLO which develop during embryogenesis, TLS develop in response to chronic inflammation and cancer, playing a crucial role in antitumor immunity by enhancing both humoral and cellular responses. Their presence, especially in intratumoral areas, has been linked to better outcomes following immunotherapy, indicating their potential as a predictive biomarker for treatment success. Determining how these biomarkers may be mechanistically linked to therapeutic response likely depends on several factors including the type of therapeutic intervention (chemotherapy, ICI, radiation, vaccine-based, ACT-based, chemokine strategies, etc.), single agent or combination therapies, sequence of combination drug administration, timepoints for biospecimen collection (Baseline, on treatment, end of treatment), TLS location and density, anatomical site, and specific cancer type. Further, these factors may differ between preclinical models and real-world settings, further complicating data interpretation and integration. Therefore, additional studies to validate TLS as a reliable biomarker for therapeutic response in OC will provide additional insights and guide future applications.

In the case of OC, several factors recently have been implicated that contribute to the complexity of the role of TLS. The anatomical site of origin of OC may contribute to not only the role that TLS play, but their potential as a biomarker for response to therapy. Cancers that develop in the OV appear to have less TLS, less B cells, and a reduced antitumor response, while TLS that originate in the FT or OM have increased TLS and more B cells, suggestive that the TME is more supportive of TLS formation and indicative of an antitumor response (125). These results not only suggest the importance of B cells in the antitumor response, but also suggest the OV has a more suppressive TME. In fact, CA-MSC present in the OV negatively impacts adaptive immunity and survival. This poses an important area for future studies to identify ways to mitigate their impact and improve TLS formation, function, and antitumor response.

Research into TLS has highlighted their influence on the balance between promoting and inhibiting tumor growth, driven by factors such as patient demographics, tumor characteristics, environmental exposures, and previous treatments. The dynamic interplay between these factors and therapeutic interventions, as well as the gut microbiome’s impact, points to the complexity of harnessing TLS for cancer therapy. While several means to induce TLS have been proposed, modulation of the microbiome through diet or probiotic efforts may be a low-cost way to affect immunity and response to ICI. Continued studies that assess fecal microbiome transplants may also be a viable option to enhance efficacy of ICI as more potential beneficial bacteria or pathways that contribute to OS in OC are identified (166).

Analysis of patients’ samples who achieved a DCB in a triple combination therapy, which included pembrolizumab, uncovered a tumor-immune-gut axis influencing immunotherapy outcomes in OC (23). Assessment of fecal biospecimens revealed several metabolites were altered in samples from DCB and LCB patients. Metabolic alterations were observed in pathways that impact immune cell function. For example, fatty acids, amino acids, indole, and purine biosynthetic pathways were altered (23). While this study had only 40 patients (21), it suggests the immune milieu and host-microbiome can be leveraged to improve antitumor response in future immunotherapy trials. Omics results identified a target, CD40 (23), that is currently in clinical trial (NCT05231122) using a CD40 agonist in combination with pembrolizumab and bevacizumab. It also suggests that strategically combining different therapies (immunotherapy, chemotherapy, PARP inhibitors, adoptive cell therapies, targeted therapies) may lead to generation of TLS and improved OS. Radiation therapy and chemotherapy have been shown to generate TLS as well. Radiation impacts infiltration of T cells and maturation of DC cells which can help drive TLS formation and reshape the TME (167). A study has shown encouraging results where neoadjuvant chemotherapy (paclitaxel and carboplatin) induced TLS formation in HGSOC suggesting that it may be used in combination with ICI to drive an anti-tumor response with increased TLS formation (168).

Several next step options to drive TLS may be through promising OC murine pre-clinical models such as the combination of cdki with ICI or use of FAK inhibitors +/- ICI. Additionally, combination therapies (ICI, chemotherapy, targeted therapy) that may modulate different components of the immune system may stimulate both the cellular and humoral response and provide a more robust DCB, as well as identify new therapeutic targets or pathways, potentially through multi-omics means. Vaccination strategies that may prime the immune response may also bolster an enhanced response through TLS generation as has been observed for PANC GM-CSF (GVAX) vaccination and HPV vaccination. CXCL13 approaches to introduce this powerful cytokine to drive TLS formation may also be a viable option in combination with ACT approaches. Finally, transcriptional assessment of different genes that may drive TLS formation may also prove to be beneficial in predicting a patient’s response by biospecimen analysis at baseline or over the course of treatment.

Advancing our knowledge of TLS, particularly in OC, and the factors affecting therapeutic response is critical. Future efforts focusing on exploiting TLS to improve immune infiltration, boost anti-tumor responses, and enhance patient responses to treatment, promise significant impact on cancer therapy optimization.
